# Developmental Changes in Food Perception and Preference

**DOI:** 10.3389/fpsyg.2021.654200

**Published:** 2021-05-18

**Authors:** Monica Serrano-Gonzalez, Megan M. Herting, Seung-Lark Lim, Nicolette J. Sullivan, Robert Kim, Juan Espinoza, Christina M. Koppin, Joyce R. Javier, Mimi S. Kim, Shan Luo

**Affiliations:** ^1^Warren Alpert Medical School of Brown University, Providence, RI, United States; ^2^Department of Pediatric Endocrinology, Hasbro Children’s Hospital, Providence, RI, United States; ^3^Department of Preventive Medicine, Keck School of Medicine of University of Southern California, Los Angeles, CA, United States; ^4^Department of Pediatrics, Keck School of Medicine of University of Southern California, Los Angeles, CA, United States; ^5^Department of Psychology, University of Missouri–Kansas City, Kansas City, MO, United States; ^6^London School of Economics, London, United Kingdom; ^7^Center for Endocrinology, Diabetes and Metabolism, Children’s Hospital Los Angeles, Los Angeles, CA, United States; ^8^Division of General Pediatrics, Children’s Hospital Los Angeles, Los Angeles, CA, United States; ^9^The Saban Research Institute of Children’s Hospital Los Angeles, Los Angeles, CA, United States; ^10^Department of Psychology, University of Southern California, Los Angeles, CA, United States; ^11^Division of Endocrinology, Keck School of Medicine of University of Southern California, Los Angeles, CA, United States

**Keywords:** eating behavior, food choice, dietary self-control, pediatric obesity, children, adolescents

## Abstract

Food choices are a key determinant of dietary intake, with brain regions, such as the mesolimbic and prefrontal cortex maturing at differential rates into adulthood. More needs to be understood about developmental changes in healthy and unhealthy food perceptions and preference. We investigated how food perceptions and preference vary as a function of age and how food attributes (taste and health) impact age-related changes. One hundred thirty-nine participants (8–23 years, 60 females) completed computerized tasks to rate high-calorie and low-calorie food cues for taste, health, and liking (preference), followed by 100 binary food choices based on each participant’s ratings. Dietary self-control was considered successful when the healthier (vs. tastier) food was chosen. Self-control success ratio was the proportion of success trials over total number of choices. Beta-weights for health (β-health) and taste (β-taste) were calculated as each attribute’s influence on food preference. Adiposity measurements included BMI z-score and waist-to-height ratio (WHtR). High-calorie foods were rated more tasty and less healthy with increasing age. Older participants liked high-calorie foods more (vs. younger participants), and β-taste was associated with age. Significant age-by-WHtR interactions were observed for health and taste ratings of high-calorie foods, β-taste, and marginally for preference of high-calorie foods. Stratifying by WHtR (high, low), we found age-related increases in taste and preference ratings of high-calorie foods in the high WHtR group alone. In contrast, age-related decreases in health ratings of high-calorie foods were significant in the low WHtR group alone. Age and β-taste were significantly associated in the high WHtR group and only marginally significant with low WHtR. Although participants rated low-calorie foods as less tasty and less healthy with increasing age, there was no association between age and preference for low-calorie foods. Participants made faster food choices with increasing age regardless of WHtR, with a significant age-by-WHtR interaction on reaction time (RT). There were no age-related effects in self-control success ratio and β-health. These results suggest that individual differences in age and central adiposity play an important role in preference for high-calorie foods, and a higher importance of food tastiness in food choice may contribute to greater preference for high-calorie foods with increasing age.

## Introduction

Every day, we make a series of dietary decisions that determine energy intake (van Meer et al., 2016; Beckerman et al., 2017). Over time, if more energy is consumed than expended, a positive energy balance is created that acts as a driver of obesity (Blundell and Cooling, 2000). The central regulation of food intake involves a delicate balance between top-down regulation from the prefrontal cortex (key region for cognitive control) and bottom-up regulation from limbic reward pathways (Lowe et al., 2020). However, during adolescence—a time of rapid brain maturation—the prefrontal cortex develops at an imbalanced, slower pace than the limbic system creating an increased risk for impaired behavioral regulation (Casey et al., 2000; Lowe et al., 2020). This is particularly relevant when adolescents are faced with appetitive cues (Somerville and Casey, 2010; Bruce et al., 2011), as well as given what is now known about continued neurodevelopment into the third decade of life (Sawyer et al., 2018). Food cues target susceptible emotions and cognitive brain functions, and can trigger automatic/habitual behaviors, particularly in children and adolescents (Berthoud, 2012; Ames et al., 2014). This knowledge can be exploited by the food industry, leading to ubiquitous food cues in our current obesogenic environment for the purposes of neuromarketing (Berthoud, 2012; Belfort-DeAguiar and Seo, 2018).

Age-related effects on the central regulation of eating have been shown in studies where younger individuals exhibit greater food craving for unhealthy foods compared to older individuals, decreased brain signal change in the prefrontal regions, and fewer connections between prefrontal-limbic regions implicated in regulation of eating (Giuliani and Pfeifer, 2015; Silvers et al., 2015; van Meer et al., 2017, 2019). Compared to adults, adolescents have been found to exhibit greater striatal sensitivity to food stimuli (Galván and McGlennen, 2013), and children exhibit greater differences between those with obesity and those with healthy weight in their response to food cues in the left insula (Morys et al., 2020).

In addition to the imbalanced development of the prefrontal cortex and limbic system, adolescents could also develop less healthy eating behaviors with increasing independence (Bassett et al., 2008; Vaitkeviciute et al., 2015). Adolescent eating habits are motivated by multiple factors including hunger and food cravings, time, convenience and availability, among many others, and they are motivated more by food preferences and food appeal, including taste, than by nutritional knowledge or perceived health benefits (Neumark-Sztainer et al., 1999; Fitzgerald et al., 2010; Lai Yeung, 2010; Naeeni et al., 2014). Food choices in adolescents are characterized by a high consumption of calorie-dense foods and low consumption of nutrient-dense foods, such as vegetables and fruits compared to children (Lytle et al., 2000; Smithers et al., 2000; Hackett et al., 2002; Mannino et al., 2004). As children get older, their dietary quality tends to decline (Smithers et al., 2000; Hackett et al., 2002), as shown in a study that compared the diet of 9–10 year old children to that of 11–12 year old children in the United Kingdom and found that the older children ate less fruits and vegetables than the younger ones (Hackett et al., 2002), as well as a National Diet and Nutrition Survey in the United Kingdom in children 4–18 years which found that potassium, magnesium and calcium intakes, as well as vitamin A levels, were lower in the older children (Smithers et al., 2000). A cohort study with third, fifth and eighth graders in Minnesota found that fruit consumption fell by 41% between the third and the eighth grades while vegetable consumption fell by 25% (Lytle et al., 2000). A study of 24-h dietary recalls in Non-Hispanic White girls ages 5, 7, and 9 years in Pennsylvania found that at age 9, significantly fewer girls were meeting the recommendations for dairy, fruit and vegetable servings than at age 5 (Mannino et al., 2004). Based on rodent studies, calorie-dense diets could alter the functional and structural maturation of the prefrontal cortex, and lead to cognitive and behavioral changes including anxiety-like behaviors, and impaired memory and decision-making (Reichelt et al., 2015, 2016, 2019; Baker and Reichelt, 2016). These alterations could have concerning long-term neurological effects in adolescents given increased neuroplasticity at this age (Lowe et al., 2020).

The observed connections between brain development and dietary intake during adolescence present an opportunity for behavioral research in food decision-making. Dietary decision-making involves integration of basic food attributes, such as tastiness, and abstract attributes, such as healthiness. Food tastiness is reliably weighted in decisions, and is a primary driver of food choices in youth 8–14 years old, as studied with a computerized food ratings and subsequent food choice (4-point scale “strong no” to “strong yes”) task (Bruce et al., 2016; Lim et al., 2016); subjects’ taste ratings predicted their food choice. Similarly, youth between the ages of 10–17 years were studied with a button-press food choice task where subjects indicated whether they wanted to eat a food by pressing “yes” or “no” (van Meer et al., 2017, 2019); subjects made their choices based on food tastiness. Food tastiness has also been shown to be a stronger predictor of food preference in older youth compared to younger youth (van Meer et al., 2019; Pearce et al., 2020). Pearce et al. (2020) studied children 7–11 years old and asked them to choose the food they preferred to eat using the computer mouse. Considering food healthiness in food decisions requires effort and is therefore unreliably weighted in decisions (Sullivan et al., 2015). In contrast to youth, however, in young adults both food tastiness and healthiness appear to contribute to food choices, even though taste still plays a more dominant role than health (Sullivan et al., 2015; Lim et al., 2018). Finally, healthy food choices require greater dietary self-control in older youth compared to younger youth, shown in a study of youth 8–13 years of age who used a computer mouse to indicate whether they wanted to eat healthy or unhealthy foods (yes or no) (Ha et al., 2016). In this study, the area under the curve (AUC) for the computer mouse actual trajectory compared to the ideal trajectory (i.e., a straight line from the start point to the selected response) was used to represent a child’s cognitive efforts to shift a decision toward the selected response, despite being initially attracted to the unselected response (Ha et al., 2016). The difference score in AUC for Yes and No curves was calculated between AUC (No choice)—AUC (Yes choice) for healthy vs. unhealthy food cues, and the AUC difference score for unhealthy foods was significantly larger than that for healthy foods, and this was most pronounced in the older children (Ha et al., 2016). Taken together, it has been shown that there is an increase in calorie-dense food consumption with increasing age, in which tastiness of food is the main determinant. In contrast, observations have been mixed in terms of effects of age on healthy food consumption and preference. We aimed to clarify whether perceptions and preferences for high-calorie and low-calorie foods vary as a function of age, and further investigate how specific food attributes (i.e., taste and health) impact these age-related changes in young individuals between 8 and 23 years old. We hypothesized that there would be an age-related increase in preference for high-calorie food items, which may be driven by the tastiness of food, with stronger integration into dietary decisions with increasing age. We did not have a clear hypothesis concerning age effects on preferences for low-calorie foods and dietary self-control given inconsistent findings in the literature. Hypotheses were generated prior to data analysis.

## Materials and Methods

### Study Participants

This was a cross-sectional study of 139 individuals between 8 and 23 years old (14.5 ± 0.42 years; 57% male). Race was reported as 79.9% Caucasian, 11.6% Asian, 2.3% African-American, and 6.2% more than one race. For ethnicity 57.4% (74/129) were Hispanic or Latino. A subset of participants had both brain structural data and food choices behavioral data, for which we published their Self-Control Success Ratio variable data (Kim et al., 2020). Data were collected for the purpose of examining how individual differences in age and adiposity influence food choices in youth, as an *a priori* research question. At the time of initiating this study and its design in 2015, there were not any similar food choices studies in youth available in the literature to utilize as a basis for power calculation. Thus, sample size was largely driven by feasibility and resources. Our data collection ended due to the Covid-19 pandemic.

In this study we included participants from age 8 to 23 years old. We included children starting at 8 years so that they would be able to understand and follow the instructions to complete the computer food ratings and food choice task, which were written at a third-grade level. Subjects 19–23 years old were included given recent proposals that the definition of adolescence be expanded to 24 years old to reflect continued neurodevelopment into the third decade of life (Sawyer et al., 2018), and given that there is a growing consensus that the age ranges studied need to span late childhood to early adulthood to assess the entire developmental period of adolescence (Foulkes and Blakemore, 2018). Participants were recruited from the pediatric endocrinology and general pediatric clinics at Children’s Hospital Los Angeles (CHLA), as well as the University of Southern California (USC) and Los Angeles community. Participants were directly approached with flyers when attending clinics or responded to flyers posted around the greater Los Angeles metro area, through previous participation in another USC research study, as well as approached at community outreach events. They were asked to participate in a study about food choices in youth. Brain imaging scans were added during the later phase of the study, and 71 participants with both brain MRI and behavioral food choices data were published in Kim et al. (2020). Inclusion criteria included: age 8–23 years, English as primary language, and being otherwise healthy. Exclusion criteria included: systemic illness, developmental delay, behavioral disorders, learning disabilities, use of psychotropic medications, and prior participation in a weight-management program. All subjects were able to read and speak English to be able to understand and respond to the computer task prompts. Parents were either Spanish- or English-speaking. Written informed consent from parents and age-appropriate assent from children were obtained. The protocol was approved by the Institutional Review Board of CHLA and USC (CHLA-15-00007 and HS-16-00978).

Parents or subjects older than 18 years old filled out a demographic questionnaire. The height and weight of all participants were measured using a stadiometer and a calibrated digital scale, respectively. Body mass index (BMI) was calculated (kg/m^2^) and BMI z-score (BMI-z) was determined based on the U.S Center for Disease Control normative data^1^. Participants aged 20 and older had BMI-z calculated for 20 years of age. Waist circumference (*n* = 136) was measured at the midpoint between the iliac crest and lower costal margin in the midaxillary line, and waist-to-height ratio (WHtR) was calculated. Average BMI-z (SE) was 0.93 ± 0.09 and WHtR was 0.52 ± 0.01.

### Computer-Based Behavioral Task

The computer-based food choice task was based on a previously published platform used to study dietary decision-making (Sullivan et al., 2015) and modified for our youth cohort with input from a pediatric dietitian and child psychologist, as previously published (Kim et al., 2020). The task consisted of a Food Ratings component and a Food Choices component, both of which were programmed in MATLAB (version 2014a, Natick, MA) with PsychophysicsToolbox (version 3)2. Instructions were designed to be at a 3rd grade reading level and were read aloud to participants who were younger than 18 years old in order to standardize for literacy. Participants completed the task in a fasted state of at least 4 h, to account for a potential desensitization to food cues in a sated state (van Meer et al., 2016). Parents were requested to wait in a separate room to control for confounding effects.

#### Food Ratings

**FIGURE 1:**
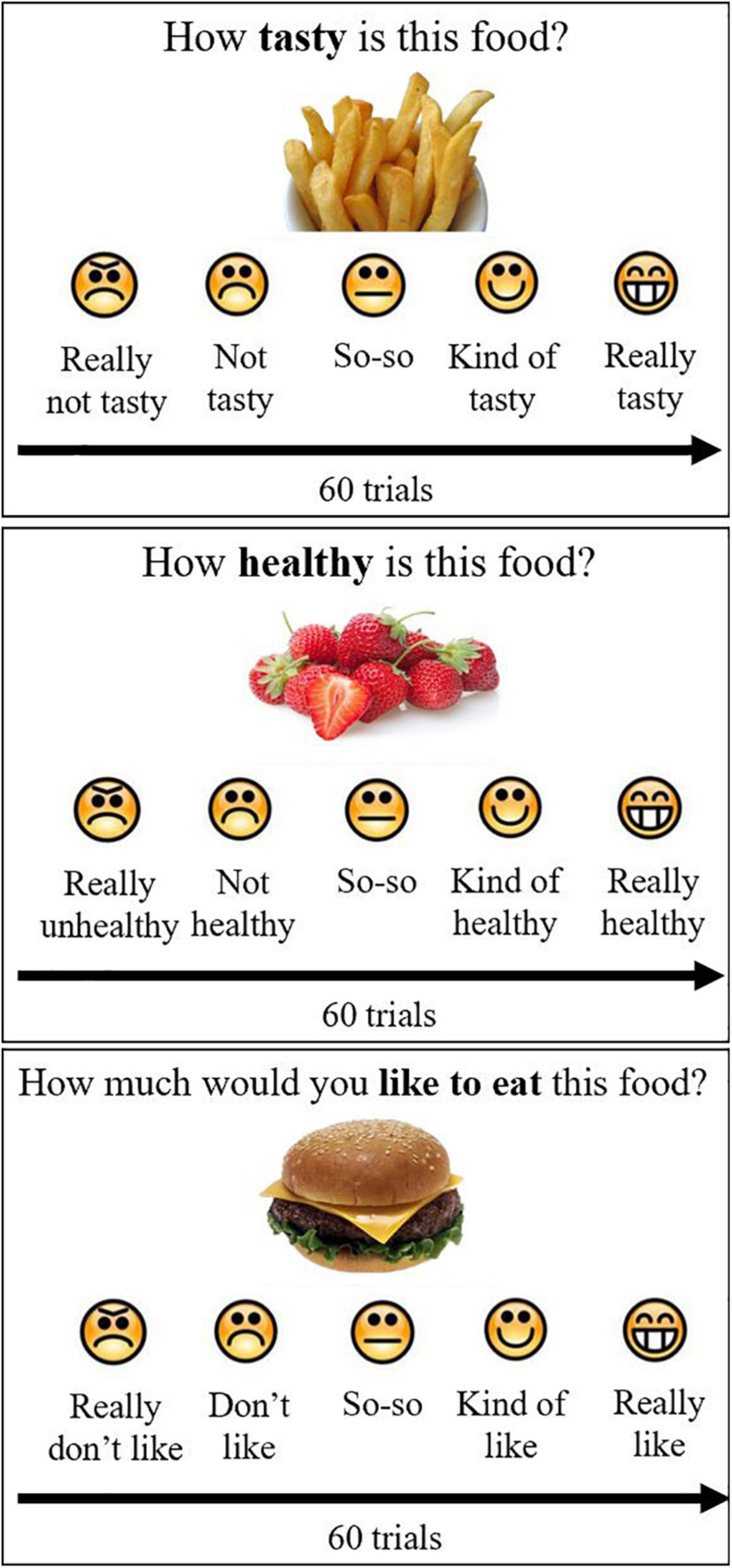
Food ratings. Participants rated 60 foods on taste, health and preference, using a 5-point scale with emoticons and words, by pushing buttons on the keyboard. Block and stimulus order were randomized across participants.

Participants were shown 30 high-calorie and 30 low-calorie food cues and were asked to rate the cues according to tastiness (“How tasty is this food?”), healthiness (“How healthy is this food?”), and preference or liking (“How much would you like to eat this food?”) (Figure 1). Block and cues order were randomized across participants. The food cue stimuli were leveraged from prior validated studies (Page et al., 2011; Blechert et al., 2014; Sullivan et al., 2015) and were matched between calorie groups for red/green/blue color proportion, size, brightness, contrast and normalized complexity so that the groups only differed by their caloric density (kcal/100 g). Caloric density for each food cue was obtained from the USDA Food Data Central^2^ and for food cues of a mixed composition was determined based on a weighted average. Food cues were selected to be foods that were familiar and appealing to a pilot group of youth. For each of the 60 cues, participants indicated their ratings (taste, health, and preference) for each food attribute on a 5-point verbal and visual Likert scale. Participants rated food tastiness as (1) really not tasty, (2) not tasty, (3) so-so, (4) kind of tasty, and (5) very tasty. Participants rated food healthiness as (1) really unhealthy, (2) not healthy, (3) so-so, (4) kind of healthy, and (5) really healthy. Participants rated preference as (1) really don’t like, (2) don’t like, (3) so-so, (4) kind of like, and (5) really like.

Based on participants’ rating of the 60 food cues, a linear regression model was created for each participant to measure how well the health rating or taste rating predicted their preference rating for food cues. A beta weight for health (β-health) and for taste (β-taste) were determined for each participant from the 60 trials to quantify the relative influence given to each attribute in determining preference for a food cue.

#### Food-Choice Mouse-Tracking

**FIGURE 2:**
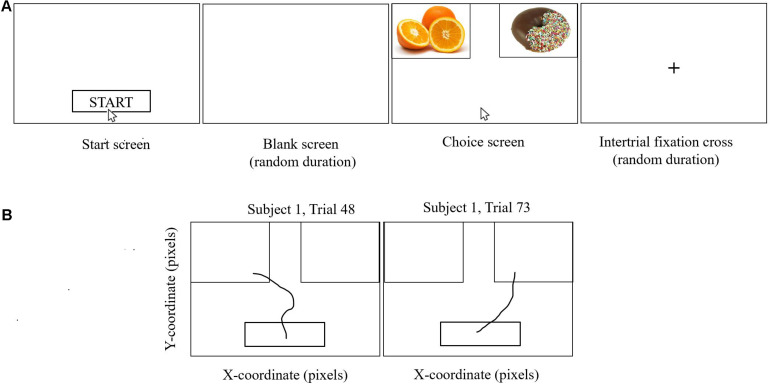
Food-choice mouse-tracking. Participants were asked to choose the food item they would like to eat. They completed several practice trials prior to starting the task. As shown in **(A)**, participants clicked on the Start button, after which there was a blank screen of random duration (200–500 ms), and then the cursor would reappear in the bottom center of the screen and the food cues appeared on the screen, one at the left upper corner and one at the right upper corner of the screen. Participants moved the computer mouse to select a response and each mouse trajectory was recorded. Once the computer mouse entered the box containing the food cue, the trial ended, without the need to click. Trajectories were not visible to the participant. Trials were separated by a fixation cross of random duration (400–700 ms). One hundred binary choices were presented to each participant based on their individual ratings for taste and health from the food ratings task. Two representative mouse paths for Subject 1 are shown in **(B)** for trials on which the left-hand food item and the right-hand food item, respectively, were selected.

The ratings were then used to construct 100 binary pairs of food cues for the participant to choose between in the food-choice mouse-tracking task (Sullivan et al., 2015; Ha et al., 2016; Lim et al., 2018). Of these 100 pairs, 75 pairs were designed to be challenge trials, wherein one of the food cues had a higher taste rating but lower health rating than the other. This task involved the participants choosing which of two food cues they would rather eat (Figure 2). Participants were reminded to “try to keep it healthy” and were told that one of their decisions would be actualized once they had completed the task. Further details on trial structure have been previously published in a subgroup of participants with brain imaging (Kim et al., 2020).

Each participant’s reaction time, the time between the presentation of the food cues and when they indicated their choice, was also calculated. A self-control success ratio, the proportion of challenge trials in which the subject chose the healthier food cue over the tastier one, was calculated for each participant.

For each subject’s mean trajectory, a multiple linear regression was constructed to predict how differences in taste ratings and health ratings between the two food cues influenced the angle of the mouse from the start point. These models were then used to find the Significance Time for health and taste per participant, defined as the time at which the respective attribute (health or taste) emerged and remained as a significant predictor of the participant’s final choice. The Significance Time was reported as the normalized time window (*t* = 1 to *t* = 101) at which a one-sided *t*-test first reported the coefficient of the particular attribute in a multiple linear regression as significantly greater than 0. The individual Significance Times were corrected by first normalizing the particular multiple linear regression coefficient of the attribute to be a proportion of its final value, and then fitting these normalized coefficients to a logistic regression; the time window at which that regression first has a non-zero value is the corrected individual Significance Time.

This recorded trajectory was analyzed to calculate the maximal deviation (MD), the furthest point of the mouse’s actual trajectory from the ideal trajectory (i.e., a straight line from the start point to the final mouse point), and the AUC, the space between the actual and ideal trajectories of the mouse’s path. MD represents how close a participant was to making a different decision while AUC is a measure of the cognitive effort with which the participants made decisions. The greater the AUC and MD, the greater the cognitive effort engaged to make a decision (Ha et al., 2016).

### Statistical Analysis

Statistical analyses were conducted using SPSS (version 27.0, Armonk, NY: IBM Corp.). Linear regressions were used to examine relationships between age and food ratings. Food ratings included taste, health and preference of high-calorie and low-calorie food cues. We additionally examined relationships between age and β-taste and β-health using linear regressions.

Mouse-tracking related variables included AUC for successful self-control trials, AUC for failed self-control trials, MD for successful self-control trials, and MD for failed self-control trials. As well, Significance Time for taste and for health. Relationships between age and self-control success ratio were investigated using linear regressions. We used linear regressions to investigate relationships between age and mouse-tracking related variables. We also examined age-related effects on reaction time during food choice using linear regressions.

Sex and WHtR were included as covariates in all the age-related models. For all the age-related models, age variable was transformed using a square-root transformation given the non-uniform distribution of age; we also explored if the quadratic model would fit the data better than the linear model of age-related effects on food ratings and food-choice mouse-tracking data. F change scores were compared between quadratic models (age + age^2^) and linear models (age). We found that F change score was not significant for all the comparisons between quadratic models and linear models, suggesting that linear models fitted data better than quadratic models in age-related effects on food ratings and food choice data.

We further examined age-by-adiposity interactions on food ratings and food-choice mouse-tracking data using linear regressions controlling for sex. We focused on WHtR as the primary adiposity measurement given it has a more pronounced effect on brain regions related to food choice than BMI-z (Kim et al., 2020; Ronan et al., 2020). When age-by-WHtR interaction was significant, we further investigated effects of age on food ratings and food-choice mouse-tracking data in high and low WHtR groups (based on the median split for WHtR of 0.51 for producing equal sample size for high and low WHtR groups) separately. Clinically, a WHtR ≥ 0.5 is considered to be a marker of central obesity and higher metabolic risk (McCarthy and Ashwell, 2006; Mehta, 2015).

A *p*-value of < 0.05 was considered to be significant for all statistical tests.

## Results

### Food Ratings

Across participants, the average rating ± SE of high-calorie foods in terms of tastiness was 3.52 ± 0.07, healthiness was 1.82 ± 0.04, and overall preference (i.e., liking) was 3.40 ± 0.07. The average rating of low-calorie foods in terms of tastiness was 3.73 ± 0.06, healthiness was 4.42 ± 0.04 and overall liking was 3.70 ± 0.06. Repeated measures of ANOVA on food ratings with two-within subject factors (calorie: high- vs. low- calorie food cues; attribute: health vs. taste) controlled for age, sex and WHtR revealed a significant main effect of calorie [*F*_(__1, 132)_ = 16.35, *p* < 0.001, partial eta squared = 0.11], such that low-calorie foods were rated higher than high-calorie foods (mean difference ± SE: 1.41 ± 0.07, *p* < 0.001). There was no significant main effect of attribute [*F*_(__1, 132)_ = 0.003, *p* = 0.96, partial eta squared = 0]. There was a significant interaction of calorie and attribute [*F*_(__1, 132)_ = 6.37, *p* = 0.013, partial eta squared = 0.046]. *Post-hoc* paired *t*-tests revealed that participants rated low-calorie foods more tasty (mean difference ± SE: 0.21 ± 0.10, *p* = 0.046) and healthier (mean difference ± SE: 2.60 ± 0.07, *p* < 0.001) than high-calorie foods. One-way ANOVA on food preference controlled for age, sex and WHtR revealed a significant main effect of calorie [*F*_(__1, 132)_ = 5.39, *p* = 0.022, partial eta squared = 0.039], such that participants liked low-calorie foods more than high-calorie foods (mean difference ± SE: 0.29 ± 0.10, *p* = 0.004).

### Food-Choice Mouse-Tracking Data

The average RT for food choices made was 1.63 ± 0.03 s. The average self-control success ratio for all participants was 33.55 ± 2.11%. Consistent with prior studies, we found that reaction time was longer during successful self-control trials than failed self-control trials (mean difference ± SE: 0.14 ± 0.04 s, *p* = 0.001). AUC and MD for successful self-control trials were larger than failed self-control trials (AUC mean difference ± SE: 1.88 ± 0.54, *p* = 0.001; MD mean difference ± SE: 0.05 ± 0.01, *p* < 0.001) suggesting that greater cognitive effort was engaged during successful self-control choices than failed self-control choices.

Across participants, the average Significance Time for taste was 899.20 ± 34.50 milliseconds (ms), and the average Significance Time for health was 1043.59 ± 33.11 ms. We also found that the Significance Times for taste were earlier than Significance Times for health (mean difference ± SE: −144.39 ± 43.88 ms, *p* = 0.001), suggesting that the taste attribute is processed earlier than health attribute in food choice.

### Effects of Age (Table 1)

To investigate age-related effects on food perceptions and food preference, we modeled relationships between age and task-related variables while controlling for sex and WHtR (Table 1).

**TABLE 1:** Summary of results for age-related effects on food ratings and food choice.

		Beta	SE	P	Effect size (partial eta squared)
Age	Taste ratings for high-calorie foods	0.49	0.11	**<0.001^∗^**	0.12
	Health ratings for high-calorie foods	−0.14	0.07	**0.03**	0.04
	Preference for high-calorie foods	0.33	0.11	**0.004**	0.06
	Taste ratings for low-calorie foods	−0.17	0.09	0.07	0.03
	Health ratings for low-calorie foods	−0.14	0.07	**0.04**	0.03
	Preference for low-calorie foods	−0.13	0.09	0.18	0.01
	β-taste	0.10	0.03	**0.002**	0.07
	β-health	−0.01	0.03	0.67	0.001
	Self-control success ratio	−0.02	0.04	0.51	0.003
	Reaction time	−0.21	0.05	**<0.001**	0.10
	Area under curve (AUC)	0.65	0.98	0.51	0.003
	Maximum deviation (MD)	−0.007	0.02	0.68	0.001
	Significance Time for taste	−1.43	3.35	0.67	0.002
	Significance Time for health	4.20	3.20	0.19	0.02
Interaction of age and waist-to-height ratio (WHtR)	Taste ratings for high-calorie foods	0.30	0.14	**0.03**	0.03
	Health ratings for high-calorie foods	−0.24	0.08	**0.003**	0.06
	Preference for high-calorie foods	0.26	0.14	0.06	0.03
	Taste ratings for low-calorie foods	−0.14	0.11	0.21	0.01
	Health ratings for low-calorie foods	−0.14	0.08	0.07	0.02
	Preference for low-calorie foods	−0.09	0.11	0.43	0.005
	β-taste	0.10	0.04	**0.01**	0.05
	β-health	−0.03	0.03	0.38	0.006
	Self-control success ratio	−0.02	0.04	0.57	0.002
	Reaction time	−0.15	0.07	**0.02**	0.04
	Area under curve (AUC)	2.51	1.16	**0.03**	0.03
	Maximum deviation (MD)	0.02	0.02	0.29	0.009
	Significance Time for taste	1.71	3.98	0.67	0.002
	Significance Time for health	3.09	3.76	0.41	0.006

*Results highlighted in bold indicate significant findings at p < 0.05.

#### Effects of Age on Food Ratings

High-calorie foods were rated as more tasty (β = 0.49, *SE* = 0.11, *p* < 0.001, partial eta squared = 0.12) and less healthy (β = −0.14, *SE* = 0.07, *p* = 0.03, partial eta squared = 0.04) with increasing age. As well, older participants indicated greater overall preference for high-calorie food cues (β = 0.33, *SE* = 0.11, *p* = 0.004, partial eta squared = 0.06) than the younger participants.

Although participants rated low-calorie foods as less tasty (β = −0.17, *SE* = 0.09, *p* = 0.07, partial eta squared = 0.03) and less healthy (β = −0.14, *SE* = 0.07, *p* = 0.04, partial eta squared = 0.03) with increasing age, there was no significant association between age and preference for low-calorie food cues (β = −0.13, *SE* = 0.09, *p* = 0.18, partial eta squared = 0.01).

Older age was associated with an increased influence of taste attribute (i.e., β-taste) on food preference (β = 0.10, *SE* = 0.03, *p* = 0.002, partial eta squared = 0.07), suggesting that the taste attribute may contribute to the age-related increases in preference for high-calorie foods. There was no significant relationship between age and β-health (β = −0.01, *SE* = 0.03, *p* = 0.67, partial eta squared = 0.001).

#### Effects of Age on Food-Choice Mouse-Tracking Data

Participants made faster food choices with increasing age (β = −0.21, *SE* = 0.05, *p* < 0.001, partial eta squared = 0.10). There was no significant association between age and self-control success ratio (β = −0.02, *SE* = 0.04, *p* = 0.51, partial eta squared = 0.003), neither were there significant associations between age and mouse-tracking related variables (AUC: β = 0.65, *SE* = 0.98, *p* = 0.51, partial eta squared = 0.003; MD: β = −0.007, *SE* = 0.02, *p* = 0.68, partial eta squared = 0.001; Significance Time for taste: β = −1.43, *SE* = 3.35, *p* = 0.67, partial eta squared = 0.002; Significance Time for health: β = 4.20, *SE* = 3.20, *p* = 0.19, partial eta squared = 0.02).

### Interactions of Age and WHtR (Table 1)

We further examined age-by-WHtR interactions on food ratings and food-choice mouse-tracking data controlling for sex.

#### WHtR, Age, and Food Ratings

**FIGURE 3:**
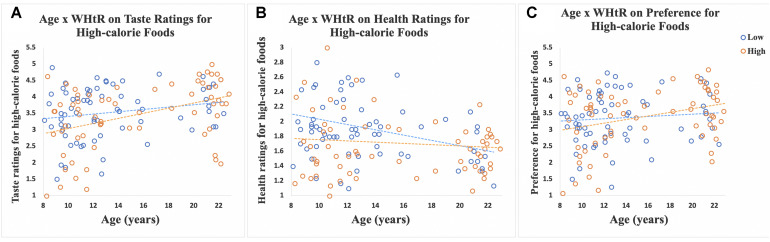
Scatterplots show ratings of 30 high-calorie and 30 low-calorie food cues by age, classified by WHtR status (categorized as high or low based on a median split for WHtR of 0.51). Data for participants with low WHtR are shown in blue circles and those with high WHtR are shown in orange circles. **(A)** Scatterplot shows the significant interaction of age-by-WHtR on taste ratings for high-calorie foods; age-related increases in taste ratings for high-calorie foods were significant in the high WHtR group but not in the low WHtR group. **(B)** Scatterplot shows the significant interaction of age-by-WHtR on health ratings; age-related decreases in health ratings for high-calorie foods were significant in the low WHtR group but not in the high WHtR group. **(C)** Scatterplot shows the marginally significant interaction of age-by-WHtR on preference ratings; age-related increases in preference for high-calorie foods were significant in the high WHtR group but not in the low WHtR group.

There was a significant interaction of age-by-WHtR on taste (β = 0.30, *SE* = 0.14, *p* = 0.034, partial eta squared = 0.03) and health (β = −0.24, *SE* = 0.08, *p* = 0.003, partial eta squared = 0.06) ratings for high-calorie foods, and a marginally significant interaction for preference ratings (β = 0.26, *SE* = 0.14, *p* = 0.06, partial eta squared = 0.03) for high-calorie foods (Figure 3). When we stratified participants into high and low WHtR groups, we found that age-related increases in taste ratings (β = 0.62, *SE* = 0.16, *p* < 0.001, partial eta squared = 0.20) and preference (β = 0.44, *SE* = 0.15, *p* = 0.006, partial eta squared = 0.11) for high-calorie food cues were significant in the high WHtR group, but not in the low WHtR group (taste ratings: β = 0.28, *SE* = 0.17, *p* = 0.11, partial eta squared = 0.04; preference ratings: β = 0.15, *SE* = 0.17, *p* = 0.40, partial eta squared = 0.01). In contrast, age-related decreases in health ratings of high-calorie foods were significant in the low WHtR group (β = −0.26, *SE* = 0.10, *p* = 0.01, partial eta squared = 0.09), but not in the high WHtR group (β = −0.06, *SE* = 0.09, *p* = 0.54, partial eta squared = 0.006).

**FIGURE 4:**
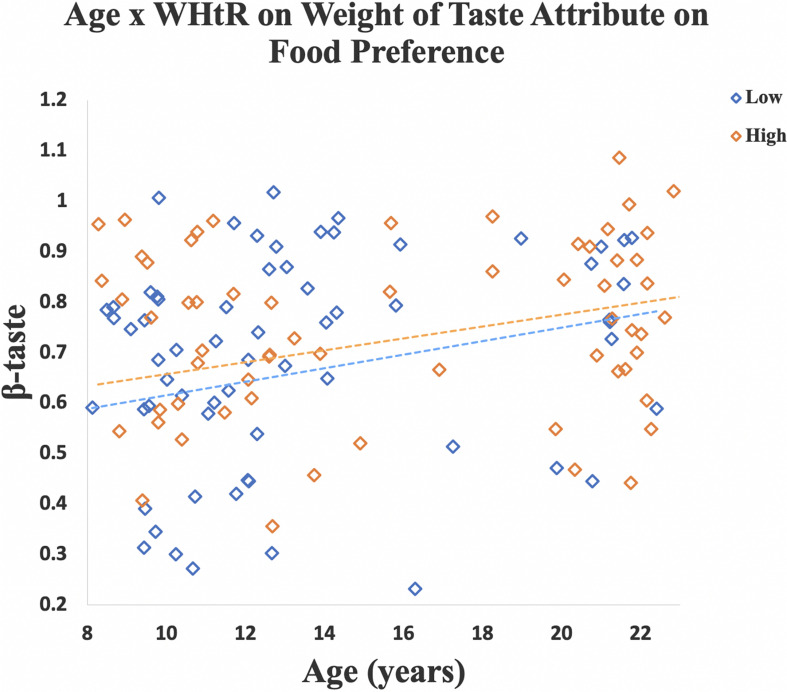
A linear regression model was created for each participant to measure how well the taste ratings predicted their preference for a specific food cue (i.e., β-weight). Scatterplot shows that older age was associated with a higher β-taste. Relationship between age and β-taste was significant in the high WHtR group, and marginally significant in the low WHtR group. Data for participants with low WHtR are shown in blue diamonds and those with high WHtR are shown in orange diamonds.

A significant age-by-WHtR interaction was observed on β-taste (β = 0.10, *SE* = 0.04, *p* = 0.01, partial eta squared = 0.05). When data were stratified into high and low WHtR groups a significant association between age and β-taste was observed in the high WHtR group (β = 0.09, *SE* = 0.04, *p* = 0.02, partial eta squared = 0.08), and a marginally significant relationship between age and β-taste was found in the low WHtR group (β = 0.10, *SE* = 0.05, *p* = 0.06, partial eta squared = 0.05) (Figure 4).

We did not find significant age-by-WHtR interactions on taste (β = −0.14, *SE* = 0.11, *p* = 0.21, partial eta squared = 0.01), health (β = −0.14, *SE* = 0.08, *p* = 0.07, partial eta squared = 0.02) and preference ratings (β = −0.09, *SE* = 0.11, *p* = 0.43, partial eta squared = 0.005) for low-calorie foods as well as β-health (β = −0.03, *SE* = 0.03, *p* = 0.38, partial eta squared = 0.006).

#### WHtR, Age, and Food-Choice

A significant age-by-WHtR interaction was observed on reaction time (β = −0.15, *SE* = 0.07, *p* = 0.02, partial eta squared = 0.04), such that both the high (β = −0.23, *SE* = 0.07, *p* = 0.003, partial eta squared = 0.13) and low (β = −0.20, *SE* = 0.09, *p* = 0.02, partial eta squared = 0.08) WHtR groups exhibited a faster reaction time with increasing age. There was a significant age-by-WHtR interaction on AUC (β = 2.51, *SE* = 1.16, *p* = 0.03, partial eta squared = 0.03). When data were stratified into high and low WHtR groups, we found no significant relationships between age and AUC in both high (β = 1.28, *SE* = 1.42, *p* = 0.37, partial eta squared = 0.01) and low WHtR (β = 0.31, *SE* = 1.40, *p* = 0.83, partial eta squared = 0.001) groups.

We did not observe significant age-by-WHtR interaction on self-control success ratio (β = −0.02, *SE* = 0.04, *p* = 0.57, partial eta squared = 0.002), MD (β = 0.02, *SE* = 0.02, *p* = 0.29, partial eta squared = 0.009), Significance Time for taste (β = 1.71, *SE* = 3.98, *p* = 0.67, partial eta squared = 0.002), and Significance Time for health (β = 3.09, *SE* = 3.76, *p* = 0.41, partial eta squared = 0.006).

## Discussion

We examined food perceptions and preferences for high-calorie and low-calorie foods in participants between 8 and 23 years old. The main finding of this study was that there was an age-related increase in preference for high-calorie food cues, particularly in those individuals with a higher WHtR. As well, we found higher taste ratings for high-calorie food cues and an increased effect of taste attribute on food preference with increasing age, especially in those with high WHtR. In contrast, health ratings for high-calorie foods declined with increasing age, particularly in those with low WHtR. Both high and low WHtR groups made faster food choices with increasing age. These findings suggest that individual differences in age and central adiposity play a more important role in preference for high-calorie foods than low-calorie foods, and that the tastiness of food may contribute to age-related increases in preference for high-calorie foods.

Consistent with other studies that demonstrate increased consumption of calorie-dense foods in adolescents (Lytle et al., 2000; Smithers et al., 2000; Hackett et al., 2002; Nielsen et al., 2002; Mannino et al., 2004; Al-Hazzaa et al., 2011), we showed an age-related increase in preference for high-calorie foods. This pattern could be explained by the differential development of the prefrontal cortex and limbic reward regions, with slower development of the prefrontal cortex, in adolescence resulting in an increased drive for rewarding behaviors (e.g., consumption of highly palatable, calorie-dense foods) and reduced cognitive regulation (Somerville and Casey, 2010; Van Leijenhorst et al., 2010; Peeters et al., 2017). We further demonstrated that age-related increases in preference for high-calorie foods were significant in individuals with a higher WHtR, but not a lower WHtR. We studied WHtR given that it is a marker of central obesity that is more directly associated with cardio-metabolic risk factors than BMI (Schneider et al., 2011; Jiang et al., 2018). We had previously reported WHtR to be related to structural brain morphology, with a negative association with prefrontal cortex thickness, and a positive association with volume of the central nucleus region of the amygdala; both regions jointly influence food choice (Kim et al., 2020). Adolescents with higher body fat vs. lower body fat have shown greater brain reward responses to food commercials, suggesting that young individuals with higher adiposity may be more responsive to appetitive food rewards (Rapuano et al., 2016). Our results further suggested that older youth with higher central adiposity are most susceptible to overconsumption of calorie-dense foods.

Our study adds to the current literature by assessing how taste and health attributes affect age-related changes in food preferences within an age-range that represents the most inclusive definition of adolescence based on neurodevelopment (Sawyer et al., 2018). With increasing age, there was a greater influence of taste attribute on food preference, suggesting that taste attribute contributes to increased preference for high-calorie foods with increasing age. These results are in line with prior studies showing that taste attribute was a stronger predictor of food preference in older vs. younger youth (van Meer et al., 2019; Pearce et al., 2020). We additionally showed an interaction of age and WHtR on β-taste, such that the group with high WHtR demonstrated a greater positive relationship between age and β-taste compared with the low WHtR group. It has been demonstrated that children with obesity, relative to children with healthy-weight, demonstrated greater responses to sweet taste (vs. water) in the insula and amygdala, regions implicated in taste processing and emotion signaling (Boutelle et al., 2015). These results suggested that youth with higher adiposity may have heightened sensitivity to appetitive taste. Our data further suggested that the association between taste and adiposity may be greater in older than younger youth. We additionally observed that older youth compared with younger ones reported higher taste ratings but lower health ratings for high-calorie foods. The former was driven by having a higher WHtR and the latter was driven by having a lower WHtR. Older youth with higher central adiposity may be most susceptible to excessive consumption of high-calorie foods that are highly palatable. It has been shown that health literacy increases with age, whereas nutritional awareness does not change as consistently with age (Naeeni et al., 2014); it has also been reported that health literacy is inversely associated with obesity in adolescents (Lam and Yang, 2014), which is consistent with our findings, with participants with low WHtR showing decreased health ratings for high-calorie foods as a function of age. This suggests it may be particularly beneficial to focus on health literacy and nutritional awareness in youth with higher abdominal obesity.

We did not find an association between age and dietary self-control, as measured by the self-control success ratio. We found the average MD was higher for choices with successful self-control than those with failed self-control, and the average AUC was also higher for successful self-control trials compared with failed self-control trials. This is consistent with increased cognitive effort when exerting self-control, yet there were no significant age-related effects on MD or AUC. While some studies showed increased cognitive effort and associated prefrontal cortex engagement during food choice or reduced craving for appetitive food cues as a function of age (Silvers et al., 2015; Ha et al., 2016; van Meer et al., 2017, 2019), we and others did not see significant age effects on dietary self-control (Pearce et al., 2020). It is generally accepted that dietary self-control is contingent on the function of the prefrontal cortex. With continued development of the prefrontal cortex into early 20s, however, dietary self-control may be still compromised in youth in the early 20s. Future studies including mapping of prefrontal cortex development and dietary self-control among individuals with a wide age-range spanning from childhood to adulthood are merited to understand the developmental trajectory of dietary self-control and prefrontal cortex development.

The strengths of the study include a sample that varied across a broad age-range, spanning the most recent definition of adolescence in terms of neurodevelopment. We used a well-designed computer task, that allows us to quantify how specific food attributes are integrated in food decision-making. There are several limitations to consider as well, including that it was a cross-sectional study, and thus causality cannot be inferred. Future longitudinal study of participants would be helpful to understand the relationships between age and preference for high- and low-calorie foods. It was challenging to recruit participants between 16 and 18 years old; future studies would benefit from enriching this specific age-range and investigate developmental trajectory of food choice behavior in the full spectrum of age. Power analysis was not performed prior to data collection, thus some null results (e.g., age effects on dietary self-control and preferences for low-calorie foods) could be due to lack of power. As well, positive results need to be interpreted with caution given there might be a possibility of type 1 error induced false positive results. There are also inherent limitations in all laboratory food choices research, as they may differ from real-life choices. Finally, future correlation of behavioral task results with brain imaging data would be useful in further understanding neurobiological underpinnings of developmental trajectory of food decision-making.

We conclude that our results are consistent with other studies that demonstrate age-related increases in consumption of calorie-dense foods in youth, in particular in those with central obesity, and suggest that age and adiposity may be more relevant to preference for high-calorie foods. Interventions targeting youth at an early age could therefore be beneficial to helping reduce consumption of high-calorie foods over time.

## Data Availability Statement

The raw data supporting the conclusions of this article will be made available by the authors, upon reasonable request.

## Ethics Statement

The studies involving human participants were reviewed and approved by the Institutional Review Board of CHLA and USC (CHLA-15-00007 and HS-16-00978). Written informed consent to participate in this study was provided by the participants’ legal guardian/next of kin.

## Author Contributions

MSG, MK, and SLu took responsibility for the integrity of the data in the study and the accuracy of the data analysis, and drafted the manuscript. SLu contributed to statistical analysis. MK, MH, and SLu provided administrative, technical, or material support, supervised the work, and obtained funding. All authors contributed to acquisition, analysis, or interpretation of data and critical revision of the manuscript for important intellectual content.

## Disclaimer

The content is solely the responsibility of the authors and does not necessarily represent the official views of the NIH, USC DORI, or the Stewart Clifton Endowment.

## Conflict of Interest

The authors declare that the research was conducted in the absence of any commercial or financial relationships that could be construed as a potential conflict of interest.

## Footnotes

1 https://www.cdc.gov/growthcharts/clinical_charts.htm

2 https://fdc.nal.usda.gov/
